# Use of ultrasound imaging software to differentiate venous and lymphatic edema in lower limbs

**DOI:** 10.1590/1677-5449.190139

**Published:** 2020-11-30

**Authors:** Vanessa Lôbo de Carvalho, Guilherme Benjamin Brandão Pitta, Sérgio Xavier Salles Cunha

**Affiliations:** 1 Universidade Estadual de Ciências da Saúde de Alagoas – UNCISAL, Departamento de Cirurgia, Maceió, AL, Brasil.; 2 Consultor em Técnicas Não Invasivas de Diagnóstico Vascular, Itanhaém, SP, Brasil.

**Keywords:** lymphedema, venous insufficiency, ultrasonics, software

## Abstract

**Background:**

Lower limb edema has both systemic and local causes. Using software to differentiate the origin of edema in ultrasound images is an innovation.

**Objective:**

To determine the parameters for using software to differentiate edema of venous and/or lymphatic origin in ultrasound images of the lower limbs.

**Method:**

This is a cross-sectional, quantitative, analytical study with non-probabilistic sampling by convenience. Data were collected by patient interview, physical examination, ultrasound examination, and analysis of software for tissue characterization in ultrasound image by means of quantification of echogenicity and Gray Scale Median (GSM).

**Results:**

The sample comprised 42 lower limbs with venous edema, 35 with lymphatic edema, 14 with mixed edema, and 11 control limbs. The distributions of pixels in echogenicity intervals by group was as follows. In the venous edema group, 88.31% were distributed from hypoechogenic interval IV to echogenic interval III; in the lymphatic edema group 71.73% were from hypoechogenic interval II to echogenic interval I; in the mixed edema group 76.17% were from hypoechogenic interval III to echogenic interval II; and in the control group 84.87% were distributed from echogenic interval II to hyperechogenic interval I. Mean and standard deviation of GSM values showed statistical differences between groups.

**Conclusion:**

The CATUS software enabled differentiation of the type of lower limb edema, facilitating diagnosis of edema type and, consequently, choice of the best therapeutic option.

## INTRODUCTION

Patients, health services, and the public health system need easy, effective, and useful tests that can measure with precision, make diagnoses, and monitor progression or remission of lymphedema,^1^ which is a disease about which little is known and on which studies are lacking. Virtual histology analysis using mode B ultrasonography (US) images can be used to quantify and characterize tissues on the basis of echogenicity brightness.^2^ Ultrasound images show echogenicity levels that are specific to each of the body’s tissues.^3^ A study by Drinan et al.^4^ states that US can be used as a method to identify dilated lymphatic vessels in the lower limbs. However, a study by Becker et al.^5^ states that it is not possible to use ultrasound to determine the cause of subcutaneous edema of the lower limbs. Neither of these studies used software to quantify pixels or brightness.

There is a need for an objective measure to quantify the changes that occur in the subcutaneous space in the presence of lymphedema. Ultrasonographic images are potentially a tool that could be used to view, assess, and quantify subcutaneous tissues in patients with lymphedema.^1^ In contrast with venous conditions, which are already widely assessed using US, there are no defined parameters for conducting a detailed assessment of lymphedema using the same examination method. This illustrates the importance of research involving US; a noninvasive and inexpensive imaging exam, for distinguishing between edema with etiology of venous and/or lymphatic origin. This study employs US and analysis of ultrasonographic images using software that performs tissue characterization by ultrasonographic imaging (CATUS), quantifying data and coloring images, discriminating between specific echogenicity levels acquired during the examination, which should facilitate interpretation by enabling differentiation of the 256 gray tones in the images, since the human eye can only see 16 tones.

Ultrasound was chosen as the underlying examination for acquiring images for assessment using the CATUS software because it is a noninvasive examination, is less expensive, and is more accessible,^6^ although the gold standard for assessment of lymphedema remains lymphoscintigraphy.^7^ The CATUS software needs US images acquired in mode B. The software’s tissue characterization is based on a study that analyzed distribution of pixels in carotid plaques in B mode US images.^3^ The study defined echogenicity intervals on a scale of 256 brightness levels as follows, blood: 0 to 4; fat: 8 to 26; muscle: 41 to 76; fibrous tissue: 112 to 196; and calcium: 211 to 255.^3^

The CATUS software has been used to assess organs, such as the kidney before and after transplant,^2^ and pathological changes, such as venous thrombosis, and can be used to classify aneurysms as acute or subacute,^8^^,^^9^ to identify thrombus in the external carotid artery,^10^ and to compare echogenic differences between limbs with lymphedema and healthy limbs.^11^ The objective of the present article is to determine the ultrasonographic image analysis software parameters for differentiation of edema of venous origin from edema of lymphatic origin in lower limbs.

## METHODOLOGY

This is a cross-sectional, observational study, with a quantitative, analytical approach, and research conducted in the field. It was approved by the Research Ethics Committee at the Universidade Federal de Alagoas, Brazil (CAAE: 58012616.5.0000.5013, CEP: 2.172.243).

The inclusion criteria for research participants were volunteers of both sexes, aged 18 or over, with edema of lower limbs diagnosed by vascular surgeons affiliated to a private hospital in the Northeast of Brazil, initially by clinical examination and then using the CATUS software to confirm the diagnosis of edema as of lymphatic and/or venous origins. Clinical diagnosis parameters were as follows: patients with lymphatic edema, defined as a specific type of edema characterized by accumulation of a fluid rich in proteins in the dermis and hypodermis;^12^ patients with venous edema, defined as edema that progresses to subcutaneous fibrosis and cutaneous atrophy in response to leakage of red blood cells and plasma proteins;^13^ and individuals without edema (control group).

The Mowlem classification was published by the American Association of Plastic Surgeons in Nashville in 1947 and was used to stratify lymphedema into three clinical phases. The first phase, Mowlem I, is characterized by a partial obstruction that will undergo complete recovery with rest and elevation for 24 to 48 hours; the second phase, or Mowlem II, involves a deficiency of the lymph vessels’ capacity for selective absorption with presence of progressive tissue fibrosis, cutaneous repercussions and shedding of skin appendages; and in the third phase, or Mowlem III, the limb swells with secondary keratinous skin abnormalities. In this phase, the lymphatics are completely surrounded by fibrosis, and the limb takes on the appearance of elephantiasis. The Mowlem classification is easy to assimilate and utilize in day-to-day practice.^14^

The CEAP (clinical [C], etiology [E], anatomy [A] and patholopysiology [P]) lower limb venous disease classification and grades for varicose veins, developed by the ad hoc committee of the American Veins Forum in 1994, was also used. The CEAP is a descriptive classification that assesses severity of venous disease and quality of life. The factors assessed are: clinical, etiology, anatomy, and pathophysiology, with the first factor subdivided into 7 classes: 0 - no visible or palpable signs of venous disease; 1 - telangiectasies or reticular veins; 2 - varicose veins; 3 - edema; 4 – trophic changes such as eczema and pigmentation; 5 - trophic changes with healed ulcer; and 6 - trophic changes with active ulcer.^15^

Individuals were excluded if they had rheumatic diseases, heart failure and/or chronic renal failure. With regard to rheumatic diseases, individuals were excluded who had suffered a lower limb trauma during the 2 months preceding the study and patients who had a prior diagnosis made by a specialist (rheumatologist and/or orthopedist).

The study assessed participants using serum sodium, creatinine, urea, potassium, and albumin assays and urinalysis to identify presence of hematuria of glomerular origin, to provide a criterion for ruling out heart failure and/or chronic renal failure. The criterion for ruling out heart failure was the New York Heart Association classification of intensity of symptoms, which has four classes. These classes stratify the degree of limitation imposed by the disease on the person’s daily activities, i.e., it is a classification that assesses a patient’s functionality and quality of life in the presence of the disease. Ratings range from absence of dyspnea symptoms during daily activities (grade I) to presence of symptoms at rest (grade IV). This stratification of symptoms by functional class has good correlations with prognosis and quality of life.^16^ Patients in classes II, III, and IV were excluded from the study.

**Figure 1:**
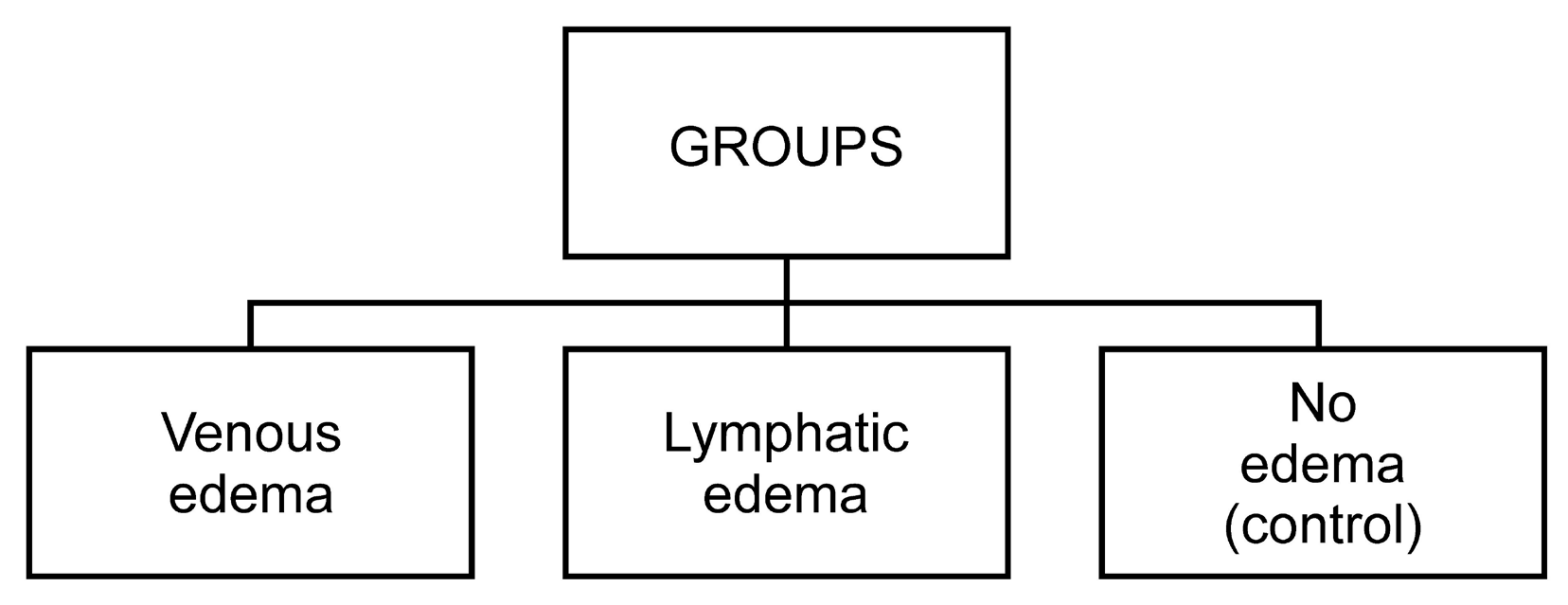
Study groups. Note: LEG = lymphatic edema group ; VEG = venous edema group; NEG = no edema group (control).

Individuals who agreed to participate were initially distributed into three groups, on the basis of their diagnoses by the hospital’s vascular surgeons. The three groups formed on the basis of the surgeons’ opinions were: group 1 – participants with lymphatic edema (LEG); group 2 - participants with venous edema (VEG); and group 3 - healthy participants with no edema, forming a control group (NEG), as shown in Figure 1.

A dedicated form was designed by the researchers to collect sample characteristics, physical examinations were conducted to determine disease characteristics, and blood and urine samples were collected for the exclusion criteria. Participants then underwent mode B US examination of the lower limbs (feet and legs), bilaterally. After acquisition of images, they were analyzed on a computer running the CATUS software.

The US examination was conducted by a single specialist who was blinded to participants’ clinical classifications, in order to standardize examinations and minimize errors, since US is an operator-dependent examination. The US unit used was a Mindray (China) M5 model with an 8-12 MHz linear transducer, with unheated standard echographic gel as the agent applied to achieve good contact between the surface of the skin and the probe. Minimal pressure was applied, to maintain the thickness and echogenicity of the region being examined.

The US unit was used in the B mode peri-vein setting (lower limb peripheral veins) which, according to the manual, was developed for studying superficial venous reflux, using a linear transducer and fan-shaped scanning. The areas investigated during the US examination were: dorsal forefoot (DF), mid-proximal leg (MPL), mid-medial leg (MML) and mid-distal leg (MDL). All areas were examined bilaterally. The linear probe was manually maintained perpendicular to the surface at four different points for each lower limb.

**Figure 2:**
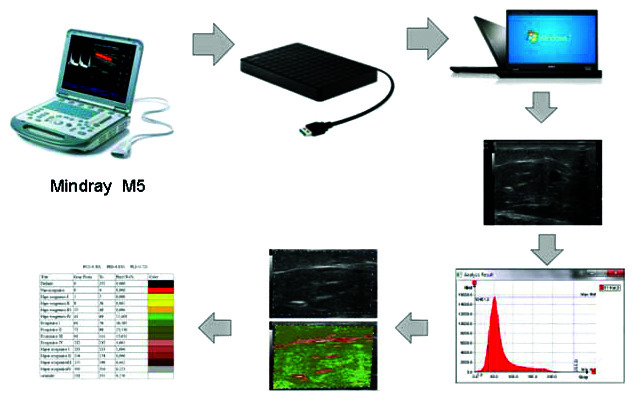
Image processing with CATUS software.

The images acquired during the US examination were copied to an external hard drive and them transferred to a computer, (Dell Inc., Texas, United States), with a core i3 processor and a Microsoft Windows 7^®^ operating system (Microsoft Corporation, New Mexico, United States), Starter version. After this transfer, the images were analyzed using the CATUS software, which had been installed on the computer in advance. Images were analyzed on CATUS individually, image by image, for each of the four predefined areas of each lower limb (Figure 2). The software can evaluate the brightness of the echogenicity levels of each imaginary unique element (pixels), attributing a value on a numerical scale (0 to 255), equating to its GSM, and coloring them according to echogenicity amplitude. The non-echogenic range is from 0 to 4; low echogenicity is from 5 to 60; echogenic is from 61 to 132; high echogenicity is from 133 to 210; and the range of saturation is from 211 to 255. Analyses using the CATUS software are an innovation in diagnosis using US images because of the ability to evaluate the distribution of pixels and brightness on the echogenicity scale, in an image with 256 grayscale tones. From this total, the human eye, unaided by technological resources, can only see 16 tones of gray. After the software has evaluated all of the grayscale tones, it colors the image to facilitate differentiation between structures that cannot be differentiated with the naked eye. Each image was analyzed according the GSM for grayscale intensities defined by Lal et al.,^3^ allocating the 256 grayscale tones around the following means: 2 for blood (0 to 4); 12 for lipids (8 to 26); 53 for muscle (41 to 76); 172 for fibrosis (112 to 196); and 221 for calcium (211 to 255).

The analyst who operated the CATUS software manually selected a bright fascia to represent level 200 along an interval of images from 0 to 255. The pixel brightness intervals for blood, fat, and calcium were adopted from Lal et al.^3^ The muscle brightness interval was subdivided into hypoechoic and hyperechoic subintervals, and the interval for fibrosis was subdivided into four subintervals: hypoechoic, part-hypo, part-hyper, and hyperechoic fibrosis. Standard brightness intervals were identified specifically as blood, fat, hypoechoic muscle, hyperechoic muscle, hyperechoic muscle fibers, and bands of calcium and fibrosis. Pixel brightness percentages were calculated for each of the 14 intervals.

Epidemiological data on lymphatic disorders were used to perform a sample size calculation for a 5% significance level, 80% test power, and a two-tailed hypothesis, resulting in a sample size of 24 individuals. Data are shown as means and standard deviations. Means were compared after applying the Levene test of homogeneity of variances. Analysis of variance (ANOVA) was used for variables with homogenous variances and the Kruskal-Wallis test was used for variables heterogeneous variances. The Tukey-HSD and Dunn post hoc tests were used as appropriate. An alpha value less than or equal to 5% was adopted for all values and calculations were performed using SPSS^®^ (IBM, New York, United States) version 21.0.

## RESULTS

**Figure 3:**
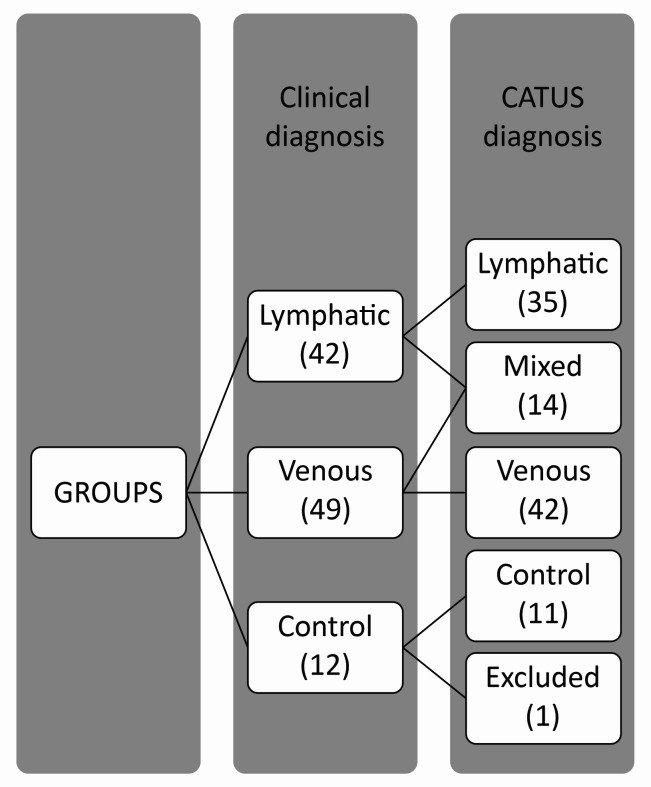
Study groups with numbers of limbs analyzed. CATUS = tissue characterization by ultrasonographic images.

The study began with three groups: 1 - LEG, with 28 participants; 2 - VEG, with 30 participants; and 3 - NEG, with 6 participants. However, as US examinations and CATUS analysis progressed, participants were identified who had both venous and lymphatic disorders causing edema, so it was necessary to create a mixed edema group (MEG). After reorganizing the study groups, there were four groups: 1 – LEG, with 25 participants and a total of 35 limbs; 2 – VEG, with 24 participants and a total of 42 limbs; 3 – NEG, with 6 participants and a total of 11 limbs; and 4 – MEG, with 9 participants and a total of 14 limbs, as shown in Figure 3.

Sample distribution by sex was as follows: VEG comprised 18 (75%) female participants and 6 (25%) males; LEG comprised 19 (76%) females and 6 (24%) males; MEG comprised 6 (66.6%) females and 3 (33.4%) males; and NEG comprised 4 (66.6%) females and 2 (33.4%) males.

In the present study, LEG had most participants with Mowlem II - 13 (52%) - and 10 with Mowlem III lymphedema (40%). These clinical phases involve fibrosis of the limb, dilatation of the lymph vessels and cutaneous changes. The first two of these findings were identified on the ultrasonographic images within areas termed “lymphatic lakes”. The VEG group had the greatest number of participants at CEAP stages 3, with 13 (54.1%), and 4, with 10 (41.6%). There was a predominance of participants with edema and trophic lesions. When the GSM and distribution of brightness of each pixel were calculated using the CATUS software, statistical differences were found between the groups.

**Figure 4:**
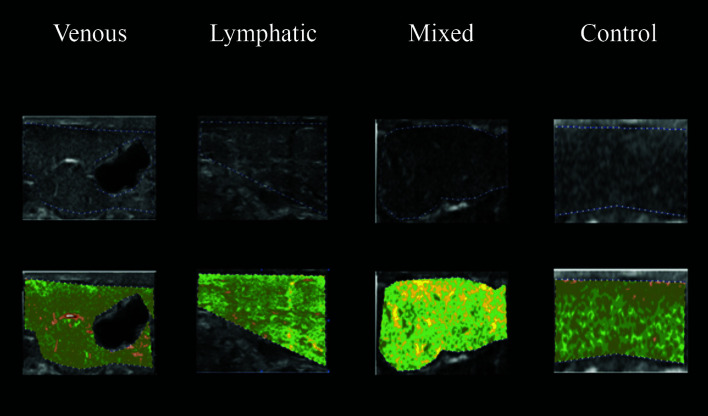
Use of CATUS software in groups, using image coloring tool after analysis of echogenicity in the MPL region. CATUS = tissue characterization by ultrasonographic images. MPL = mid-proximal leg

The ultrasonographic images from the four study groups were analyzed using CATUS and histograms were generated by the software with the echographic images. All participants were analyzed with CATUS for all four regions of the leg. In the ultrasonographic images from LEG, “lymphatic lakes” were visible, and granular edema could be seen in images from VEG patients with venous edema. The MEG images had both “lymphatic lakes” and granular edema, as shown in Figures 4 [Fig gf0500] [Fig gf0600]-7. The entire VEG group had reflux in at least one vein of the lower limbs examined. The “lymphatic lakes” were hypoechogenic and their GSM predominantly comprised pixels in the echogenicity range of 0 to 40, i.e., from non-echogenic to echogenic III.

**Figure 5 gf0500:**
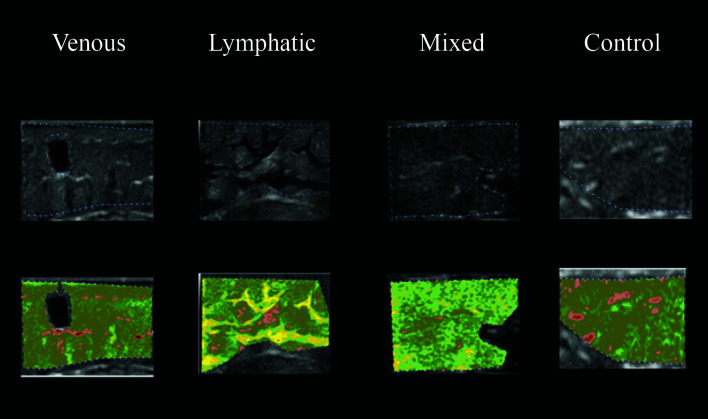
Use of CATUS software in groups, using image coloring tool after analysis of echogenicity in the MML region. CATUS = tissue characterization by ultrasonographic images. MML = mid-medial leg

**Figure 6 gf0600:**
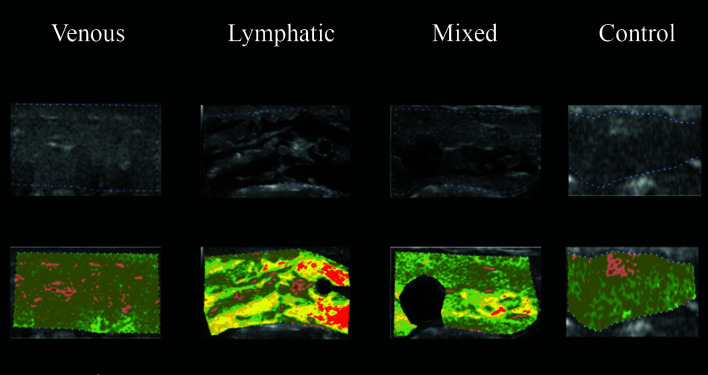
Use of CATUS software in groups, using image coloring tool after analysis of echogenicity in the MDL region. CATUS = tissue characterization by ultrasonographic images.

**Figure 7:**
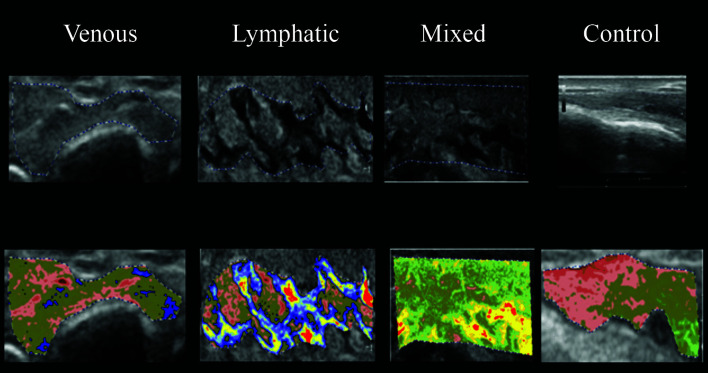
Use of CATUS software in groups, using image coloring tool after analysis of echogenicity in the DF region. CATUS = tissue characterization by ultrasonographic images. DF = dorsal forefoot

The distribution of pixels across echogenicity intervals by group was as follows. In the venous edema group, 88.31% were distributed from hypoechogenic interval IV to echogenic interval III; in the lymphatic edema group, 71.73% were from hypoechogenic interval II to echogenic interval I; in the mixed edema group, 76.17% were from hypoechogenic interval III to echogenic interval II; and in the control group, free from edema, 84.87% were distributed from echogenic interval II to hyperechogenic interval I.

## DISCUSSION

As lymphedema progresses, the protein content of the interstitial liquid increases with cellular infiltration and sometimes tissue fibrosis develops and fat accumulates.^17^ This fibrosis occurs in the skin, in the subcutaneous tissues, and in lymphatic collectors. Over the course of its progression, lymphedema provokes changes in the region where chronic tissue edema occurs, including inflammation, fibrosis, and fat build up; however, it is not yet fully clear whether these changes are involved in the cause or simply indicate worse clinical status.^18^ Correct diagnosis makes it possible to take the appropriate therapeutic decisions with regard to compression and prophylactic care for the skin.^19^

Chronic venous stasis provokes cutaneous changes, such as stasis dermatitis, hyperpigmentation, lipodermatosclerosis, athrophie blanche, and venous ulcers.^19^ Chronic venous insufficiency in lower limbs can provoke pain, edema, cutaneous changes with hyperpigmentation, fibrosis, and ulceration.^20^ Venous edema in the lower limbs is, generally, the first clinical sign of venous insufficiency, appearing in the region of the malleolus and disappearing when the patient is at rest.^21^ In turn, edema has the potential to cause significant injury, starting with inadequate nutrition of the subcutaneous tissue and muscles, provoking pain and potentially progressing to venous ulcer.^22^

In US images, the lymph vessels are generally seen as small hypoechoic circles that show the internal lumen. If the lymph vessels are normal, i.e., are not dilated, the lumen is too small to be seen on US. In contrast, dilated lymph vessels are easily detected because they have a lumen from 0.5 to 1.0 mm in diameter.^23^ Suehiro et al.^17^ state that dilated lymphatics can be seen, but they consider it is difficult to identify dilated lymphatics exclusively by identification of subcutaneous spaces oriented vertically. Normal lymph vessels cannot be identified on US, whether without analysis or with analysis with the CATUS software, since it is designed to quantify the brightness amplitude of each pixel by the characteristic echogenicity of the structure and normal vessels have an area that is too small to stand out in ultrasonographic images. Nevertheless, visualization of the vertically aligned subcutaneous spaces that are present in lymphedema is facilitated by CATUS in the areas with “lymphatic lakes”.

The CATUS software analyzes images using the brightness of each pixel, enabling closer comparisons with pathological histology.^9^ The software provides the GSM and the distribution of the brightness of each pixel along the grayscale corresponding to echogenicity of ultrasonographic images, enabling structures to be identified by quantification of pixels at a given level of echogenicity, for tissue characterization and for distinguishing the origin of edema in the lower limbs.

Accumulated lymph increases the proportion of adipose tissue and the volume of the area involved, preventing lymph pumping and affecting the skin, setting up a cycle that makes it difficult to cure the disease. Adipose tissue is lobulated and has a fibrous septal network, typical of the complexity in this region, termed a “lymphatic lake” in this study. Limbs with lymphedema are of varying sizes and may have large adipocytes, in contrast with areas of normal limb, where adipocytes are of uniform size.^24^ The compromised lymphatic drainage can influence distribution of fat in obese patients.^25^ These elements, such as adipose tissue and fibrosis, are easily identified by CATUS, using the previously defined echogenicity levels.

The increased free space in the superficial fascia and the echogenicity of subcutaneous tissue is a nonspecific finding that has no utility for characterizing the different types of lower limbs edema. Regardless, high-resolution US is a widely available resource that is safe, inexpensive, and noninvasive, and is recommended as an aid to clinical diagnosis and treatment decision in lower limb edema. Ultrasonographic images also offer the advantage that they can be shared with patients and other professionals.^26^

Low-resolution US can be used for staging of secondary lower limb lymphedema on the basis of echogenicity, providing an objective representation of the severity of lymphedema. Characteristics such as changes to skin and subcutaneous tissue are seen in chronic lymphedema, caused by extracellular changes such as connective tissue hypertrophy, build-up of fat caused by hypertrophy and hyperplasia of adipocytes and interstitial disorders, such as accumulation of protein-rich fluids.^23^

A study comparing a healthy lower limb with one with lymphedema identified differences based on the presence of channels or “lymphatic lakes” in the limb with lymphedema and absence of them in the healthy limb. These differences, also observed in this study, were observed in analyses conducted using CATUS to quantify echogenicity, which was lower in the lymphatic extremity, due to the presence of the lymphatic “lakes” and/or channels, which were hypoechogenic compared to the healthy limb.^11^

Phan, Cherry, and Ryan^22^ conducted a study with 12 patients with venous ulcers in the lower limbs, analyzing the dermis using mode B US. They found that dermal echogenicity was significantly reduced, indicating accentuated edema of the papillary dermis compared with controls. The present study analyzed venous edema and not specifically the dermis in this type of edema. The data found indicated a predominance of pixels in the echogenic region corresponding to the band above the interval greater than 40 in the area of edema, which corresponds to muscle and fibrosis.

Iker et al.^27^ conducted a study evaluating echogenicity in the dermis of 12 limbs of patients with lipedema and ten limbs of patients with lymphedema. They found that the mean dermal echogenicity at the ankle was 68 on the brightness scale in patients with lymphedema. Those data are similar to the findings of the present study, in which there was a predominance of hypoechogenic areas in the region affected by the lymphedema, corresponding to brightness levels 41 to 60 on the grayscale, although cutaneous US was not conducted.

CATUS offers reliable measures that are useful for identifying the origin of edema, whether venous and/or lymphatic, and can therefore be used as a tool to aid the choice of therapeutic intervention. The method can be used to make a diagnosis and assess disease severity, and also for objectively assessing response to treatment. Notwithstanding, the imaging exam currently used for assessing lymphatic edema is lymphoscintigraphy, which is an expensive and inaccessible examination compared to US, in addition to having the disadvantage of being invasive. It is therefore necessary to conduct comparative studies of diagnosis of lymphedema using US imaging and CATUS analysis with lymphoscintigraphy.

## CONCLUSIONS

This study offers an evaluation of a new examination for lymphedema, which is widely accessible to the population, noninvasive, inexpensive, and safe, transforming a well-known imaging exam into a more objective assessment. This is achieved by quantification of pixels and brightness on the echogenicity scale, facilitating reproducibility and objectivity in analysis of ultrasonographic images. This new form of image analysis should have major economic and social impacts, facilitating accessibility and choice of the best therapeutic option.

This study serves as a foundation for further studies using CATUS, including of applications in other parts of the body, such as the upper limbs, bearing in mind the growing incidence of breast cancer and its possible sequelae, including upper-limb lymphedema. There is a need for more studies using CATUS to follow-up the same patient, assessing a range of treatments and their impacts in patients with venous and/or lymphatic edema. It is also necessary to conduct multicenter research using CATUS and seek to identify correlations with other biochemical markers that have not yet been studied in patients with edema of venous and/or lymphatic origin.

## References

[1] Johnson KC, DeSarno M, Ashikaga T, Dee J, Henry SM (2016). Ultrasound and clinical measures for lymphedema. Lymphat Res Biol.

[2] Valiente Engelhorn AL, Engelhorn CA, Salles-Cunha SX, Ehlert R, Akiyoshi FK, Assad KW (2012). Ultrasound tissue characterization of the normal kidney. Ultrasound Q.

[3] Lal BK, Hobson RW, Pappas PJ (2002). Pixel distribution analysis of b-mode ultrasound scan images predicts histologic features of atherosclerotic carotid plaques. J Vasc Surg.

[4] Drinan KJ, Wolfson PM, Steinitz D (1993). Duplex imaging in Lynfedema. J Vasc Tech..

[5] Becker M, Schilling T, Von Beckerath O, Kröger K (2015). Sonography of subcutaneous tissue cannot determine causes of lower limb edema. Vasa Bern..

[6] Suehiro K, Morikage N, Yamashita O (2017). Differentiation of functional venous insufficiency and leg lymphedema complicated by functional venous insufficiency using subcutaneous tissue ultrasonography. J Vasc Surg Venous Lymphat Disord.

[7] Hassanein AH, Maclellan RA, Grant FD, Greene AK (2017). Diagnostic accuracy of lymphoscintigraphy for lymphedema and analysis of false-negative tests. Plast Reconstr Surg Glob Open.

[8] Cassou-Birckholz MF, Engelhorn CA, Salles-Cunha SX (2011). Assessment of deep venous thrombosis by grayscale median analysis of ultrasound images. Ultrasound Q.

[9] Salles Cunha SXS (2012). Nota técnica: avaliação ultrassonográfica de aneurismas da aorta tratados com endopróteses. J Vasc Bras.

[10] Barros FS, Pontes SM, Prezotti BB, Sandri GA, Salles-Cunha SX, Barros FS (2013). Trombo móvel na carótida interna: planejamento cirúrgico definido pela ultrassonografia vascular. Arq Bras Cardiol Imagem Cardiovasc..

[11] Carvalho V, Salles-Cunha SX, Braga F (2018). Ultrasonographic, quantitative comparison of lower extremity lymphedema versus normal control: technical note with case reports. Veins and Lymphatics..

[12] Naouri M, Samimi M, Atlan M (2010). High-resolution cutaneous ultrasonography to differentiate lipoedema from lymphoedema. Br J Dermatol.

[13] França LHG, Tavares V (2003). Insuficiência venosa crônica. Uma atualização. J Vasc Bras.

[14] Perez MDCJ, Guedes HE, Belczak CEQ (2009). Classificação dos linfedemas. Linfologia: diagnóstico, clínica e tratamento..

[15] Eklöf B, Rutherford RB, Bergan JJ (2004). Revision of the CEAP classification for chronic venous disorders: consensus statement. J Vasc Surg.

[16] Bocchi EA, Marcondes-Braga FG, Bacal F, Ferraz AS, Albuquerque D, Rodrigues D (2009). III Diretriz Brasileira de Insuficiência Cardíaca Crônica. Arq Bras Cardiol.

[17] Suehiro K, Morikage N, Yamashita O (2016). Distribution of extracellular fluid in legs with venous edema and lymphedema. Lymphat Res Biol.

[18] Zampell JC, Yan A, Elhadad S, Avraham T, Weitman E, Mehrara BJ (2012). CD4+ Cells Regulate Fibrosis and Lymphangiogenesis in Response to Lymphatic Fluid Stasis. PLoS One.

[19] Cambria RA, Gloviczki P, Naessens JM, Wahner HW (1993). Noninvasive system with prospective, 386 evaluation of the lymphatic lymphoscintigraphy: A semiquantitative analysis in extremities. J Vasc Surg.

[20] Lattimer CR, Kalodiki E, Geroulakos G, Hoppensteadt D, Fareed J (2016). Are Inflammatory Biomarkers Increased in Varicose Vein Blood?. Clin Appl Thromb Hemost.

[21] Hu D, Phan TT, Cherry GW, Ryan TJ (1998). Dermal o edema assessed by high frequency ultrasound in venous leg ulcers. Br J Dermatol.

[22] Hall JE, Guyton AC (2011). Guyton & Hall: Tratado de Fisiologia Médica.

[23] Hara H, Mihara M (2017). Usefulness of preoperative echography for detection of lymphatic vessels for lymphaticovenous anastomosis. SAGE Open Med Case Rep.

[24] Tashiro K, Feng J, Wu SH (2017). Pathological changes of adipose tissue in secondary lymphoedema. Br J Dermatol.

[25] Mortimer OS, Rockson SG (2014). New developments in clinical aspects of lymphatic disease. J Clin Invest.

[26] Suehiro K, Morikage N, Murakami M, Yamashita O, Samura M, Hamano K (2013). Significance of ultrasound examination of skin and subcutaneous tissue in secondary lower extremity Lymphedema. Ann Vasc Dis.

[27] Iker E, Mayfield CK, Gould DJ, Patel KM (2019). Characterizing lower extremity lymphedema and lipedema with cutaneous ultrasonography and an objective computer-assisted measurement of dermal echogenicity. Lymphat Res Biol.

