# Supplementary material for: Use of ultrasound imaging software to differentiate venous and lymphatic edema in lower limbs
Source: J Vasc Bras. 2020 Nov 30;19:e20190139. doi: 10.1590/1677-5449.190139 (PMC8217995; doi:10.1590/1677-5449.190139)
Supplement: Translation

# Translation: Uso de *software* na imagem ultrassonográfica para diferenciação de edema de origem venosa e de origem linfática em membros inferiores

**Authors:** Vanessa Lôbo de Carvalho, Guilherme Benjamin Brandão Pitta, Sérgio Xavier Salles Cunha

**Affiliations:** 1 Universidade Estadual de Ciências da Saúde de Alagoas – UNCISAL, Departamento de Cirurgia, Maceió, AL, Brasil.; 2 Consultor em Técnicas Não Invasivas de Diagnóstico Vascular, Itanhaém, SP, Brasil.

**Keywords:** linfedema, insuficiência venosa, ultrassom, software

## Abstract

**Contexto:**

O edema de membros inferiores (MMII) possui causas de origem sistêmicas e locais. Uma inovação é a utilização de um *software* de caracterização tecidual para diferenciação da origem do edema em imagens de ultrassom.

**Objetivos:**

Determinar os parâmetros do uso de *software* na imagem ultrassonográfica para diferenciação de edema de origem venosa e/ou linfática em MMII.

**Métodos:**

Trata-se de um estudo transversal do tipo quantitativo analítico com amostragem não probabilística por conveniência. Os instrumentos de coleta foram: anamnese, exame físico, exame de ultrassom e análise do *software* de caracterização tecidual por imagem ultrassonográfica por meio da quantificação da ecogenicidade e da mediana da escala de cinza (GSM).

**Resultados:**

A amostra foi composta por 42 MMII de edema venoso, 35 de edema linfático, 14 de edema misto e 11 controle. Quanto à distribuição dos pixels nos intervalos de ecogenicidade, o grupo com edema venoso apresentou 88,31% entre o intervalo hipoecogênico IV ao ecogênico III; o grupo com edema linfático, 71,73% entre o intervalo hipoecogênico II ao ecogênico I; grupo com edema misto, 76,17% entre o intervalo hipoecogênico III ao ecogênico II; e o grupo controle, 84,87% entre o intervalo ecogênico II ao hiperecogênico I. A média e o desvio-padrão da GSM apresentaram diferença estatística entre os grupos.

**Conclusão:**

O *software* CATUS permitiu a diferenciação do tipo de edema de MMII, facilitando o diagnóstico do tipo de edema e, consequentemente, a escolha da melhor opção terapêutica.

## INTRODUÇÃO

Os pacientes, os serviços de saúde e o sistema de saúde pública necessitam de testes fáceis, eficientes e úteis, que mensurem com precisão, façam o diagnóstico e o acompanhamento da progressão ou regressão do linfedema^1^, doença pouco conhecida ou com carência de estudos. A histologia analisada virtualmente por meio da imagem da ultrassonografia (US) modo B permite a quantificação e a caracterização de tecido com base no brilho de ecogenicidade^2^. As imagens de US são apresentadas por ecogenicidade específicas a cada tecido corporal^3^. O estudo de Drinan et al.^4^ afirma que é possível utilizar a US como método para identificar linfáticos dilatados em membros inferiores (MMII). Todavia, o estudo de Becker et al.^5^ afirma que não é possível, sonograficamente, determinar a causa de edema subcutâneo de MMII. Ambos os estudos citados anteriormente não utilizaram nenhum *software* para a quantificação de pixels e de brilhos.

É desejável uma medida objetiva para quantificar as mudanças que ocorrem no espaço subcutâneo na presença de linfedema. A imagem ultrassonográfica é uma ferramenta potencial para visualizar, avaliar e quantificar o tecido subcutâneo em pacientes com linfedema^1^. Diferente das lesões venosas, que já são amplamente avaliadas por meio de US, o linfedema não possui parâmetros definidos para uma avaliação minuciosa pelo mesmo exame. Isso ratifica a importância de pesquisas que envolvam a US, exame de imagem não invasivo e de fácil acesso, para uma distinção da etiologia do edema de origem venosa e/ou linfática. A presente pesquisa utiliza a US e a análise das imagens ultrassonográficas por meio do *software* que realiza a caracterização tecidual por imagem ultrassonográfica (CATUS) ao quantificar os dados e colorizar, discriminando determinadas ecogenicidades obtidas durante o exame, o que facilita a sua interpretação ao permitir a diferenciação dos 256 tons de cinza que existem na imagem, já que o olho humano só consegue visualizar 16 tons.

A escolha da US como exame de base para avaliação das imagens pelo *software* CATUS é justificada pelas características de ser um exame não invasivo, menos oneroso e mais acessível^6^, sendo o exame de padrão-ouro para avaliar o linfedema - a linfocintilografia^7^. O *software* que realiza a CATUS necessita das imagens do exame de US modo B. Esse *software* baseou sua caracterização tecidual em uma pesquisa que analisou a distribuição de pixels na placa carotídea por meio de US modo B. Este estudo definiu os intervalos de ecogenicidade para sangue 0 a 4, gordura, 8 a 26, músculo, 41 a 76, fibra, 112 a 196 e cálcio, 211 a 255, tendo 256 amplitudes de brilho^3^.

O *software* CATUS já avaliou órgãos, como o rim, antes e após transplante^2^, alterações patológicas, como trombose venosa e pode classificar aneurismas como agudos ou subagudos^8^^,^^9^, identificar trombo na artéria carótida externa^10^ e fazer a comparação de diferenças ecogênicas entre o membro com linfedema e o sadio^11^. O objetivo do presente artigo é determinar os parâmetros do *software* na imagem ultrassonográfica para diferenciação de edema de origem venosa e de origem linfática em MMII.

## METODOLOGIA

Trata-se de um estudo de natureza observacional e transversal, de abordagem quantitativa e analítica, com pesquisa de campo. Essa pesquisa foi aprovada pelo Comitê de Ética em Pesquisa da Universidade Federal de Alagoas, CAAE 58012616.5.0000.5013, CEP 2.172.243.

Os critérios de inclusão dos participantes na pesquisa foram voluntários de ambos os sexos e com idade mínima de 18 anos, sendo portadores de edema em MMII com diagnóstico fornecido por médicos cirurgiões vasculares pertencentes a serviço de hospital privado do nordeste do Brasil, inicialmente por meio de exame clínico e, posteriormente, através do *software* CATUS, que confirmou o diagnóstico do edema como de causa linfática e/ou venosa. Foram utilizados como parâmetro do diagnóstico clínico: pacientes com edema linfático definido como tipo específico de edema que se caracteriza por um acúmulo de fluido rico em proteínas na derme e na hipoderme^12^; pacientes com edema venoso, definido como edema que progride para fibrose subcutânea e atrofia cutânea em resposta às hemácias e proteínas plasmáticas extravasadas^13^; e indivíduos sem edema (grupo controle).

Para o linfedema, utilizou-se a classificação de Mowlem, publicada pela Associação Americana de Cirurgiões Plásticos em Nashville em 1947, para estratificá-lo em três fases clínicas. A primeira fase, ou Mowlem I, se caracteriza por uma obstrução parcial que apresenta recuperação completa com repouso e elevação de 24 a 48 horas; a segunda fase, ou Mowlem II, tem a capacidade de absorção seletiva dos vasos linfáticos deficientes com a presença de fibrose progressiva tecidual, repercussões cutâneas e queda de fâneros; na terceira fase, ou Mowlem III, há o alargamento do membro com alterações queratóticas secundárias na pele. Nessa fase, os linfáticos estão completamente envolvidos pela fibrose, e o membro se encontra com o aspecto elefantiásico. A classificação de Mowlem é de fácil assimilação e uso na prática diária^14^.

Utilizou-se também a classificação e graduação da doença venosa dos MMII [CEAP, classificação clínica (C), etiológica (E), anatômica (A) e patológica (P)] para varizes que foi desenvolvida pelo comitê *ad hoc* do Fórum Americano de Veias em 1994. A CEAP é uma classificação descritiva que avalia a severidade da doença venosa e a qualidade de vida. Os fatores avaliados são: clínica, etiologia, anatomia e patologia, sendo o primeiro fator subdividido em 7 classes: 0 - sem sinais visíveis ou palpáveis de doença venosa; 1 - telangiectasias ou veias reticulares; 2 - veias varicosas; 3 - edema; 4 - alterações tróficas, como eczema e hipercromia; 5 - alterações tróficas com úlcera cicatrizada; e 6 - alterações tróficas com úlcera aberta^15^.

Foram excluídos os indivíduos portadores de doenças reumáticas, insuficiência cardíaca e/ou renal crônica. Quanto às doenças reumáticas, foram excluídos os indivíduos que sofreram algum traumatismo nos MMII nos 2 meses que antecederam a pesquisa e os pacientes que possuíam o diagnóstico anterior à pesquisa por médicos especialistas (reumatologista e/ou ortopedista).

A pesquisa avaliou os participantes por exames séricos de sódio, creatinina, ureia, potássio e albumina e sumário de urina para identificar a presença de hematúria de origem glomerular a fim de servir como critério de exclusão de insuficiência cardíaca e/ou de insuficiência renal crônica. Quanto à insuficiência cardíaca, foi utilizado como critério de exclusão a categorização da intensidade de sintomas proposta pela classificação da New York Heart Association, que a divide em quatro classes. Essas classes estratificam o grau de limitação imposto pela doença para atividades cotidianas do indivíduo, ou seja, é uma classificação que avalia a funcionalidade e a qualidade de vida do paciente frente a sua doença. Ela avalia desde a ausência de sintomas de dispneia durante atividades cotidianas (grau I) até a presença de sintomas no repouso (grau IV). Essa estratificação de sintomas pela classe funcional possui uma boa correlação com o prognóstico e qualidade de vida^16^. O presente estudo excluiu pacientes pertencentes às classes II, III e IV.

Os indivíduos que aceitaram participar foram distribuídos em três grupos inicialmente, conforme a avaliação e o diagnóstico dos cirurgiões vasculares do hospital. Os três grupos formados, segundo os pareceres dos médicos, foram: 1 - grupo de participantes com edema linfático (GEL); 2 - grupo de participantes com edema venoso (GEV); e 3 - grupo de participantes saudáveis para grupo controle (GSE), conforme a Figura 1.

**Figura 1:** Divisão dos grupos da pesquisa. Nota: GEL = grupo de edema linfático; GEV = grupo de edema venoso; GSE = grupo sem edema/controle.

Foram realizados o preenchimento de um formulário elaborado pelos pesquisadores para caracterização da amostra, o exame físico para caracterização da doença e a coleta do exame de sangue e de urina para critérios de exclusão. Posteriormente, foram submetidos a US do modo B em MMII na região de pé e pernas bilateralmente. Após a obtenção das imagens, elas foram analisadas pelo *software* CATUS, em um computador.

O exame de US foi realizado por um único especialista que não conhecia a classificação clínica dos participantes, a fim de padronizar o exame e minimizar erros, já que a US é um exame que depende do operador. O aparelho de US utilizado foi da marca Mindray (China), modelo M5 com transdutor linear de 8-12 MHz, com gel ecográfico padrão não aquecido como agente de acoplamento entre a superfície da pele e a sonda. Uma mínima pressão foi aplicada para preservar a espessura e a ecogenicidade da região estudada.

O aparelho de US foi utilizado no modo B no tipo peri-veia (venoso periférico das extremidades inferiores) que, segundo o manual do aparelho, foi criado para estudar o refluxo venoso superficial, com o transdutor linear e com a varredura em forma de leque. As áreas pesquisadas durante o exame de US foram: região dorsal do antepé (DA), região médio-proximal da perna (PMP), região médio-medial da perna (PMM) e região médio-distal da perna (PMD). Todas as áreas foram avaliadas bilateralmente. A sonda linear foi mantida manualmente e perpendicular à superfície em quatro locais diferentes para cada MMII.

As imagens coletadas no exame de US foram copiadas no HD externo e transferidas para um computador da marca Dell Inc. (Texas, EUA), com processador i3core e sistema operacional Microsoft Windows 7^®^ (Microsoft Corporation, Novo México, EUA), versão Starter. Após essa transferência, as imagens foram analisadas pelo *software* CATUS, previamente instalado no computador. A análise de imagem através da CATUS ocorreu de forma individual, sendo analisada imagem por imagem nas quatro áreas predefinidas nos MMII (Figura 2). Esse *software* permitiu a avaliação da amplitude do brilho nas ecogenicidades dos elementos imaginários únicos (pixels), que são atribuídos a uma escala numérica (0 a 255), da GSM e da colorização das amplitudes de ecogenicidade. A área não ecogênica se estende de 0 a 4; baixa ecogenicidade, 5 a 60; ecogênico, 61 a 132; alta ecogenicidade, 133 a 210; e saturado, 211 a 255. As análises no *software* CATUS surgem como uma inovação em diagnóstico por meio de imagens de US, em virtude de sua capacidade de avaliar a distribuição dos pixels e do brilho na escala de ecogenicidade, em uma imagem que possui 256 tons de cinza. Desse total, o olho humano sem recursos tecnológicos só visualiza 16 tons de cinza. O *software*, após avaliação dos tons de cinza, coloriza a imagem para facilitar a diferenciação de estruturas que, a olho nu, não é possível. A imagem foi analisada pela GSM da intensidade da escala de cinza conforme Lal et al.^3^, que dispõe os 256 tons de cinza com sua média: de 2 para sangue (0 a 4), 12 para lipídios (8 a 26), 53 para músculo (41 a 76), 172 para fibrose (112 a 196) e 221 para cálcio (211 a 255).

**Figura 2:** Processamento de imagem para *software* CATUS.

O analista que utilizou o *software* CATUS selecionou manualmente uma fáscia brilhante para representar o nível 200 de um intervalo de imagens de 0 a 255. Os intervalos de brilho do pixel para o tipo de sangue, de gordura e de cálcio foram realizados de acordo com o estudo de Lal et al.^3^. O intervalo de brilho muscular foi subdividido em intervalos hipoecoicos e hiperecoicos, enquanto o da fibra foi subdividido em quatro intervalos: fibra hipo, meio-hipo, médio-hiper e hiperecogênica. Os intervalos de brilho padrão foram especificamente identificados em sangue, gordura, músculo hipoecoico, músculo hiperecoico, fibras musculares hiperecoicas e faixas de cálcio e fibra. As porcentagens de brilho de pixels foram calculadas para cada um dos 14 intervalos.

A partir de dados epidemiológicos sobre distúrbios linfáticos, foi realizado o cálculo do tamanho da amostra, com nível de significância de 5%, com poder do teste de 95% e com hipótese bicaudal, a qual estipulou o tamanho da amostra de 24 indivíduos. Os dados estão apresentados como média e desvio-padrão. Realizou-se a comparação entre médias após o teste de homogeneidade das variâncias (Levene). As variáveis com variâncias homogêneas foram submetidas ao teste estatístico de análise de variância (ANOVA), enquanto as com heterogeneidade das variâncias foram submetidas ao teste de Kruskal-Wallis. Os testes *post hoc* utilizados foram o Tukey-HSD e o Dunn, respectivamente. Para todos os valores, adotou-se um valor de alfa menor ou igual a 5% e o pacote estatístico SPSS^®^ (IBM, Nova Iorque, EUA) da versão 21.0.

## RESULTADOS

A pesquisa iniciou com três grupos: 1 - GEL, com 28 participantes selecionados; 2 - GEV, com 30 participantes; e 3 - GSE, com 6 participantes. Todavia, durante a realização da US e da análise da CATUS, foram identificados participantes com lesões venosas e linfáticas como causa do edema, sendo necessário criar o grupo de edema misto (GEM). Após a reorganização dos grupos da pesquisa, ficaram quatro grupos: 1 – GEL, com 25 participantes e total de 35 membros; 2 – GEV, com 24 participantes e total de 42 membros; 3 – GSE, com 6 participantes e total de 11 membros; e 4 – GEM, com 9 participantes e total de 14 membros, conforme mostra a Figura 3.

**Figura 3:** Divisão dos grupos da pesquisa com quantidade de membros. CATUS = caracterização tecidual por imagem ultrassonográfica.

Na amostra quanto ao sexo, obteve-se o seguinte resultado: o GEV apresentou 18 (75%) participantes do sexo feminino e 6 (25%) do masculino; o GEL apresentou 19 (76%) do sexo feminino e 6 (24%) do masculino; o GEM apresentou 6 (66,6%) do sexo feminino e 3 (33,4%) do masculino; e o GSE apresentou 4 (66,6%) do sexo feminino e 2 (33,4%) do masculino.

Na presente pesquisa, o GEL apresentou um maior número de participantes com linfedema Mowlem II com 13 (52%) e Mowlem III com 10 (40%). Essas fases clínicas apresentam a fibrose do membro, a dilatação dos vasos linfáticos e a alteração cutânea. As duas primeiras alterações foram identificadas nas áreas analisadas denominadas de “lagos linfáticos” na imagem ultrassonográfica. O grupo GEV obteve maior número de participantes no CEAPs 3, 13 (54,1%) e 4, 10 (41,6%). Houve o predomínio dos participantes com a presença de edema e alteração trófica. Ao se calcular a GSM e a distribuição da amplitude de brilho de cada pixels por meio do *software* CATUS, identificou-se que houve diferença estatística entre os grupos.

Os quatro grupos pertencentes à presente pesquisa tiveram suas imagens ultrassonográficas analisadas por meio da CATUS e os histogramas gerados pelo *software* com o ecográfico. Todos os participantes foram analisados pela CATUS nas quatro regiões da perna predefinidas no estudo. Nas imagens ultrassonográficas do GEL, houve a presença de “lagos linfáticos”, e no GEV, de um edema granular nos pacientes com edema venoso. O GEM apresentou “lagos linfáticos” e edema granular, conforme as Figuras 4 [Fig gf05] [Fig gf06]-7. O grupo GEV apresentou em sua totalidade o refluxo de alguma veia nos MMII pesquisados. Os “lagos linfáticos” apresentaram a característica de serem hipoecogênicos e possuírem uma GSM com predomínio de pixels nas faixas de 0 a 40 na ecogenicidade, ou seja, do não ecogênico ao ecogênico III.

**Figura 4:** Uso do *software* CATUS nos grupos com recurso de colorização da imagem após análise de ecogenicidade na região PMP. CATUS = caracterização tecidual por imagem ultrassonográfica; PMP = região médio-proximal da perna.

**Figura 5 gf05:** Uso do *software* CATUS nos grupos com recurso de colorização da imagem após análise de ecogenicidade na região PMM. CATUS = caracterização tecidual por imagem ultrassonográfica; PMM = região médio-medial da perna.

**Figura 6 gf06:** Uso do *software* CATUS nos grupos com recurso de colorização da imagem após análise de ecogenicidade na região PMD. CATUS = caracterização tecidual por imagem ultrassonográfica; PMD = região médio-distal da perna.

**Figura 7:** Uso do *software* CATUS nos grupos com recurso de colorização da imagem após análise de ecogenicidade na região DA. CATUS = caracterização tecidual por imagem ultrassonográfica; DA = região dorsal do antepé.

Quanto à distribuição dos pixels nos intervalos de ecogenicidade, o grupo com edema venoso apresentou 88,31% entre o intervalo hipoecogênico IV e o ecogênico III; o grupo com edema linfático, 71,73% entre o intervalo hipoecogênico II e o ecogênico I; o grupo com edema misto, 76,17% entre o intervalo hipoecogênico III e o ecogênico II; e o grupo sem edema, 84,87% entre o intervalo ecogênico II e o hiperecogênico I.

## DISCUSSÃO

Quando o linfedema progride, o líquido intersticial aumenta em teor de proteína, com infiltração celular, eventualmente desenvolvendo fibrose tecidual e deposição de gordura^17^. Essa fibrose ocorre na pele, nos tecidos subcutâneos e nos coletores linfáticos. O linfedema provoca alterações na região que apresenta o edema tecidual crônico ao longo do seu desenvolvimento, como inflamação, fibrose e deposição de gordura; todavia, ainda não está esclarecido se essas alterações têm causalidade ou apenas indicam a piora do quadro clínico^18^. O diagnóstico adequado permite a melhor escolha terapêutica de terapia de compressão e cuidados profiláticos com a pele^19^.

A estase venosa de forma crônica provoca alterações cutâneas como dermatite de estase, hiperpigmentação, lipodermatosclerose, atrofia branca e úlceras venosas^19^. A insuficiência venosa crônica de MMII pode provocar dores, edema, alterações cutâneas com hiperpigmentação, fibrose e ulceração^20^. O edema venoso em MMII é, geralmente, o primeiro sinal clínico de insuficiência venosa, aparecendo na região do maléolo e desaparecendo no decorrer do repouso^21^. Esse edema, por sua vez, tem um potencial de lesão grande, que se inicia através de uma nutrição inadequada do tecido subcutâneo e dos músculos, provocando dor e podendo avançar para uma úlcera venosa^22^.

No estudo com US, os vasos linfáticos são visualizados geralmente como pequenos círculos hipoecoicos que indicam que o lúmen interno. Caso os vasos linfáticos estejam normais, ou seja, não estiverem dilatados, o lúmen é muito pequeno para ser observado com o exame de US. Já os vasos linfáticos dilatados são facilmente detectados por ter um lúmen entre 0,5 a 1,0 mm^23^. Em seu estudo, Suehiro et al.^18^ afirmaram que podem ser identificados linfáticos dilatados, embora considerem difícil a identificação de linfáticos dilatados somente por meio da observação de espaços subcutâneos orientados verticalmente. Os vasos linfáticos normais não são identificados pela US sem a análise nem após a análise com o *software* CATUS, já que o mesmo se propõe a quantificar a amplitude de brilho em cada pixel por ecogenicidade característica da estrutura e esses vasos normais apresentam uma área muito pequena que não se destaca na imagem ultrassonográfica. No entanto, a visualização dos espaços subcutâneos orientados verticalmente, que existem na presença do linfedema, é facilitada com a CATUS nas regiões denominadas “lagos linfáticos”.

No *software* CATUS, as imagens são analisadas por meio da amplitude dos brilhos de cada pixel, possibilitando a comparação com a histologia patológica de forma mais aproximada^9^. O *software* fornece a GSM e a distribuição da amplitude de brilho de cada pixel na escala de cinza correspondente à ecogenicidade da imagem ultrassonográfica, permitindo a realização de identificação da estrutura analisada por meio da quantificação de pixels em determinada ecogenicidade para caracterização tecidual e distinção da origem do edema de MMII.

A linfa acumulada ocasiona o aumento do tecido adiposo e do volume da área acometida, dificultando o bombeamento linfático e afetando a pele, o que gera um ciclo que dificulta a cura da doença. O tecido adiposo possui lobulação e uma rede septal fibrosa, o que caracteriza a complexidade nessa região, denominada de “lago linfático” no presente estudo. O membro com linfedema possui tamanhos variados, inclusive com a presença de grandes adipócitos, em contraste com áreas de membros normais que possuem adipócitos com tamanho uniforme^24^. A drenagem linfática prejudicada pode influenciar a disposição da gordura na presença de obesidade^25^. Os elementos citados anteriormente, como tecido adiposo e fibrose, são facilmente identificados pela CATUS com as ecogenicidades previamente estabelecidas.

O aumento dos espaços livres nas fáscias superficiais e ecogenicidade no tecido subcutâneo é um achado inespecífico e inútil para caracterizar os diferentes tipos de edema de MMII. Todavia, a US com alta resolução é um recurso amplamente disponível, seguro, barato e não invasivo, sendo recomendada para auxiliar no diagnóstico clínico e na escolha terapêutica de edemas de MMII. As imagens ultrassonográficas ainda apresentam a vantagem de poderem ser compartilhadas com pacientes e outros profissionais^26^.

A US de baixa resolução avalia o estágio do linfedema secundário de MMII por meio da ecogenicidade para representar objetivamente a gravidade do linfedema. Características como as alterações na pele e do tecido subcutâneo são encontradas no linfedema crônico, decorrentes da alteração extracelular como hipertrofia do tecido conjuntivo, acúmulo de gordura resultante da hipertrofia e hiperplasia de adipócitos e de alterações intersticiais, como o acúmulo de fluidos ricos em proteínas^23^.

Um estudo comparativo entre um MMII sadio e um com linfedema apontou para diferenças com base na presença de canais ou “lagos linfáticos” no membro com linfedema e ausência deles no membro sadio. Essas diferenças, observadas neste estudo, foram obtidas com a análise por meio da CATUS, a qual quantificou a ecogenicidade, que se apresentou menor na extremidade linfática devido à presença dos “lagos” e/ou canais linfáticos que se apresentaram hipoecogênicos em comparação com o membro sadio^11^.

Phan, Cherry e Ryan^22^ realizaram um estudo com 12 pacientes portadores de úlceras venosas em MMII, analisando a derme deles por meio de US modo B. Identificaram que a ecogenicidade dérmica foi significativamente reduzida, indicando um edema acentuado da derme papilar em comparação com os pacientes controles. O presente estudo avaliou o edema venoso e não especificamente a derme nesse tipo de edema. Os dados encontrados apontaram para o predomínio de pixels na região ecogênica correspondente à faixa superior ao intervalo superior a 40 na região do edema, que corresponde a músculo e fibrose.

No estudo de Iker et al.^27^, foi avaliada a ecogenicidade da derme de 12 membros de pacientes portadores de lipedema e dez membros de pacientes portadores de linfedema. Identificou-se que a ecogenicidade dérmica média no tornozelo foi de 68 na escala de brilho em pacientes com linfedema. Tais dados aproximaram-se dos obtidos no presente estudo, por haver predomínio de áreas hipoecogênicas na região afetada pelo linfedema que corresponde ao brilho de 41 a 60 na escala de cinza, apesar de não ter sido realizada uma US cutânea nesta pesquisa.

A CATUS é uma medida de resultado confiável e útil para identificar a origem do edema, seja venoso e/ou linfático, servindo como ferramenta para escolha da melhor intervenção terapêutica. O método pode fazer o diagnóstico e avaliar a gravidade da doença, bem como aferir objetivamente a resposta à terapia. Todavia, atualmente, o exame de imagem para avaliação do edema linfático é a linfocintilografia, exame oneroso e pouco acessível comparado à US, além de ter a desvantagem de ser invasivo. Diante disso, é necessário realizar um estudo comparativo entre o diagnóstico por imagem de linfedema com a imagem da US e análise CATUS e com a linfocintilografia.

## Conclusão

A presente pesquisa permite a avaliação de um novo exame para linfedema, de fácil acesso para a população, não invasivo, de baixo custo e seguro, transformando um exame de imagem já conhecido em uma avaliação mais objetiva. Isso é realizado por meio da quantificação de pixels e brilho na escala de ecogenicidade, que facilita a reprodutibilidade e objetividade da análise da imagem ultrassonográfica. Essa nova forma de avaliação de imagem trará grande impacto econômico e social, facilitando a acessibilidade e a escolha da melhor opção terapêutica.

Esta pesquisa serve de base para novos estudos utilizando a CATUS, inclusive em outras áreas do corpo, como no membro superior, haja vista o crescente número de câncer de mama e suas possíveis sequelas, a exemplo do linfedema de membro superior. Há necessidade de serem realizadas mais pesquisas com a CATUS para o acompanhamento do mesmo paciente, avaliando diversas terapêuticas e seus impactos nos pacientes com edema venoso e/ou linfático. Além disso, é necessário realizar pesquisas multicêntricas utilizando a CATUS e buscando correlações com outros dados bioquímicos até agora não estudados em portadores de edema de origem venosa e/ou linfática.
